# Sex ratio disparities in the two most common cancers worldwide: an exploratory analysis using GLOBOCAN 2022 data, gender inequalities, and economic indicators

**DOI:** 10.1016/j.eclinm.2026.103855

**Published:** 2026-04-02

**Authors:** Amalia Martinez, Léna Bonin, Pascale Grosclaude, Michelle Kelly–Irving, Cyrille Delpierre, Sébastien Lamy

**Affiliations:** a Equity Team, CERPOP UMR 1295 Inserm Université de Toulouse, Toulouse, France; b Research Team Accredited by the French National League Against Cancer, France; c Institut Claudius Regaud, Institut Universitaire du Cancer de Toulouse – Oncopole, Toulouse, France; d Registre des Cancers du Tarn, Institut Claudius Regaud, Albi, France

**Keywords:** Gender inequalities, Economic context, Sex ratio of cancer incidence, Lung cancer, Colorectal cancer

## Abstract

**Background:**

Lung and colorectal cancers are the most common non-sex-specific cancers worldwide and represent a major share of global incidence and mortality. Although international variations in incidence are well documented, the contribution of structural gender inequalities and economic context to these disparities remains insufficiently explored. This study explores how gender inequalities and economic context relate to male-to-female incidence sex ratios of lung and colorectal cancers in a subset of 29 countries.

**Methods:**

We conducted an ecological analysis using GLOBOCAN 2022 data. The study included 29 countries with high-quality and comparable data on gender inequality, economic indicators, and cancer incidence. Associations between the Gender Inequality Index (GII), gross domestic product (GDP) per capita, and incidence sex ratios were examined using multivariable linear regression models. Sensitivity analyses were performed to assess robustness, including exclusion of influential countries and log-transformed models.

**Findings:**

Structural gender inequalities, measured by the GII, were strongly associated with higher male-to-female incidence sex ratios for lung cancer, and this relationship persisted after adjustment for smoking prevalence. This suggests that social and structural mechanisms beyond behavioural risk factors contribute to sex disparities in lung cancer incidence. For colorectal cancer, economic context was the principal determinant of sex differences in incidence, while associations with gender inequality were limited and variable. In more gender-equal, high-income countries, colorectal cancer incidence tended to converge between men and women, likely reflecting increasingly similar exposures and lifestyles.

**Interpretation:**

These findings highlight the importance of integrating structural gender inequalities and macroeconomic context into analyses of cancer incidence. Incorporating such social determinants alongside behavioural and biomedical risk factors may improve the relevance of prevention strategies and cancer surveillance efforts, particularly in high-income settings. However, these findings should be interpreted with caution given the ecological design of the study and the limited ability to account for all potential confounding or mediating factors.

**Funding:**

This project was funded by the Gender and Health Inequalities (GENDHI) project, ERC-2019-SyG grant agreement No. 856478.

**Research in context**

**Evidence before this study**

Sex differences in cancer incidence have been widely documented, particularly for lung and colorectal cancers, the most common non–sex-specific cancers worldwide. Previous studies have largely attributed these disparities to individual behavioural risk factors such as tobacco use, alcohol consumption, diet, and occupational exposures. A search of PubMed and major epidemiological literature up to 2024 using terms related to *gender inequality*, *sex differences*, *cancer incidence*, *lung cancer*, and *colorectal cancer* identified studies primarily examining individual behavioural determinants or socioeconomic context separately. Most existing research therefore focuses on individual-level risk factors, whereas the potential role of structural gender inequality in shaping cancer incidence patterns across countries remains underexplored. However, little evidence exists on whether structural indicators of gender inequality are associated with cross-national variations in cancer incidence sex ratios, particularly when considered alongside economic development.

**Added value of this study**

This ecological study explores whether male-to-female incidence ratios for lung and colorectal cancers vary according to structural indicators of gender inequality and economic development across 29 high-income countries with high-quality cancer registry data. Unlike previous research focussing on individual behavioural or socioeconomic risk factors, our analysis demonstrates that cross-national variations in cancer incidence sex ratios are associated with broader structural factors. By adopting a life-course perspective and using early exposure indicators, we highlight how structural gendered inequities can shape population-level disparities in cancer incidence, providing a complementary lens to understand sex differences beyond individual behaviours.

**Implications of all the available evidence**

Together with existing literature, these findings suggest that male-to-female differences in cancer incidence cannot be understood solely through biological or individual behavioural factors. Structural social and economic contexts, such as gender inequality and national economic development, also shape sex-specific cancer patterns. Integrating these indicators into population-based cancer research may support the development of more equitable prevention strategies and improve the global understanding of cancer disparities. However, these findings should be interpreted with caution given the ecological design of the study and the limited ability to account for all potential confounding or mediating factors.

## Introduction

Cancer is a major public health issue and the second leading cause of death worldwide, with nearly 20 million new cases in 2022. Globally, cancer incidence is about 14% higher in men (212.5 per 100,000 people) than in women (186.0 per 100,000 people), although these figures include sex-specific cancers.^1^ Among non-sex-specific cancers, lung and colorectal cancers are the leading causes of incidence and mortality in both sexes, yet they exhibit marked sex differences. In 2022, the global male-to-female incidence ratio was approximately 2.0 for lung cancer and 1.4 for colorectal cancer.^1^^,^^2^

These disparities reflect both behavioural differences and broader structural factors.^3^, ^4^, ^5^ Gender norms and social roles shape exposure to risk factors, men are more frequently exposed to tobacco, alcohol, and occupational carcinogens,^5^^,^^6^ while women, despite broader healthcare engagement, may face socioeconomic barriers that limit access to prevention and diagnosis.^6^^,^^7^ Beyond individual behaviours, gender operates as a structural system shaping life-course opportunities and access to health resources.^8^ The Gender Inequality Index (GII) captures several dimensions of these structural inequalities at the country level, including reproductive health (maternal mortality, adolescent birth rate), empowerment (education level and parliamentary representation), and labour market participation.^9^

Economic context also affects cancer incidence through healthcare infrastructure, early detection and living conditions. Gross Domestic Product (GDP) per capita is widely used as a proxy for national economic development and its potential investment in population health. Although GDP does not capture resources are allocated or the equity of access to care, it remains a pragmatic for international comparisons. In cross-country analyses, GDP per capita is often categorised to better capture differences in economic context and to identify threshold effects or non-linear associations with cancer outcomes.^10^^,^^11^

Both gender inequality and economic context influence international variations in lung and colorectal cancer incidence.^12^^,^^13^ Advances in screening, diagnosis and treatment can exacerbate inequalities: low- and middle-income countries face later diagnoses and higher mortality, including gendered disparities in access.^14^

Although previous research has examined gender inequality and economic context separately, no ecological study to date has jointly analysed their independent and combined influence on sex differences in cancer incidence across countries.

This study addresses this gap by exploring how GII and GDP per capita are associated, independently and jointly, with male-to-female incidence ratios for lung and colorectal cancers. For lung cancer, we hypothesised that higher GII would be associated with greater male over-incidence and that higher GDP/capita would be linked to narrower sex gaps, given the stronger gendered behavioural patterns associated with this cancer. For colorectal cancer, we expected both higher GII and higher GDP/capita to be associated with lower male-to-female incidence ratios, although with weaker associations due to less gender-specific risk factors.

In this study, “sex” refers to the male and female categories reported in cancer registry data, whereas “gender” refers to structural and societal inequalities captured by the GII.

## Methods

**Fig. 1:**
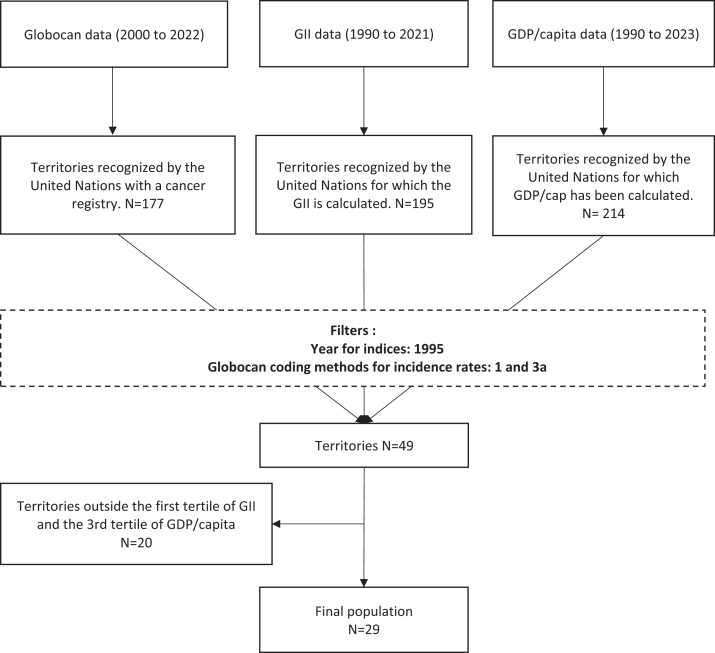
Flowchart of countries selection for analysis. **Note:** The flowchart summarises country selection based on data availability and quality criteria for GLOBOCAN incidence (https://gco.iarc.who.int/today/en/data-sources-methods-by-country-detailed?tab=8), the UNDP Gender Inequality Index (1995) (https://hdr.undp.org/data-center/thematic-composite-indices/gender-inequality-index#/indicies/GII), and World Bank GDP per capita (1995). Full inclusion and exclusion criteria are described in the Methods. **GLOBOCAN data quality codes: Code 1:** High-quality national or regional cancer registry data (>50% population coverage). **Code 3a:** High-quality regional cancer registry data (<50% population coverage) combined with validated modelling.

Data sources and variables (Fig. 1).

### Ethical approval

This study used publicly available aggregated data and did not involve individual-level human participants. Therefore, ethical approval was not required.

### Main outcome

Cancer incidence data were extracted from GLOBOCAN 2022 (IARC), providing age- and sex-stratified annual incidence rates per 100,000, based on national or subnational cancer registries and statistical models. Only countries with high-quality data (Codes 1 and 3a) were included.^15^ Rates were age-standardised (Segi-Doll reference). Analyses focused on lung (ICD-10 C33–C34) and colorectal cancer (ICD-10 C18–C20). The primary outcome was the male-to-female incidence rate ratio.

### Main exposures

Gender inequality was measured using the Gender Inequality Index (GII, 1995), a composite score ranging from 0 (equality) to 1 (maximum inequality). GII was standardised and used as a continuous variable. Values correspond to UNDP's retrospectively harmonised estimates for that year.

Economic context was assessed using GDP per capita (GDP/capita, 1995),^16^ expressed in thousands of US dollars. GDP was standardised and categorised into three quartile-based groups (Low: ≤ Q1; Medium: Q1-Q3; High: ≥ Q3 [reference]), following recommendations for international analyses of socioeconomic indicators.^10^^,^^11^

The year 1995 was selected for both indicators to account for the latency between structural exposures and cancer incidence, reflecting a life-course perspective.

### Statistical analysis

Analyses were conducted in RStudio (2023.12.1 + 402). To enhance comparability and limit confounding by outliers, analyses focused on countries in the lowest tertile of GII and the highest tertile of GDP/capita, ensuring high economic development, low gender inequality, and reliable cancer data.

Both GII and GDP/capita were standardised (mean-centred, and scaled by one standard deviation (SD)) to improve model stability and allow comparison of effect sizes.^17^ Coefficients thus represent the effect of a one-SD increase in each predictor.

### Regression modelling

Associations between gender inequality, economic context, and cancer incidence sex ratio were assessed using multiple linear regressions. Four models were fitted for each cancer site, with indicator variables for Medium- and Low-GDP groups (High GDP as reference):

• Model 1: GII only

Sex ratio = β0+β1⋅GII+ε.

• Model 2: GDP/capita only

Sex ratio = β0+β1⋅Medium_GDP+β2⋅Low_GDP+ε.

• Model 3: GII and GDP/capita

Sex ratio = β0+β1⋅GII+β2⋅Medium_GDP+β3⋅Low_GDP+ε.

• Model 4: Model 3+interaction

Sex ratio = β0+β1⋅GII+β2⋅Medium_GDP+β3⋅Low_GDP+β4⋅(GII × Medium_GDP)+ β5⋅(GII × Low_GDP)+ε.

Predicted values and 95% confidence intervals were calculated for each GDP group and visualised with fitted lines and confidence bands, alongside observed data to illustrate the distribution across the GII gradient.

Multicollinearity was evaluated using variance inflation factors (VIF). In models including only main effects (Model 3), VIF values were below conventional thresholds, indicating moderate collinearity. In interaction models (Model 4), VIF values exceeded recommended thresholds, reflecting strong collinearity between GII and GDP categories (Tables 2 and 3).

### Residual diagnostics and sensitivity analyses

Residual diagnostics included graphical checks for homoscedasticity and normality (residual-fitted plots, Q–Q plots). Influential, outlier and leverage observations were identified using Cook's distance (threshold: 4/n), studentized residuals, and DFBETAs.^18^ Countries exceeding a Cook's distance threshold of 0.14 were considered influential (Appendix 1 and 5).

For sensitivity analyses, the most influential country in each model was excluded, and all regression models and visualisations were re-estimated. Full results are presented in Appendix 2 (lung cancer) and Appendix 6 (colorectal cancer). GDP/capita was also tested as a continuous predictor in Models 2 and 3 to assess linearity.

To further assess model robustness, the incidence sex ratio was also analysed on a logarithmic scale. Re-estimating the four models with log (sex ratio) did not change the direction or interpretation of the associations, nor improve model fit. Linear models were therefore retained for simplicity and interpretability.

### Complementary analyses

To account for major risk factors influencing sex ratios in cancer incidence, additional analyses were performed where data were available. For lung cancer, the sex ratio of smoking prevalence (tobacco use in 2000, WHO Global Health Observatory) was included in supplementary models to assess its impact on the GII/GDP–incidence sex ratio relationship.

For colorectal cancer, comparable international, sex-disaggregated data on key risk factors (e.g., red meat consumption) were not available for most countries, preventing parallel analyses. Regarding organised colorectal cancer screening, official information was identified for only 14 countries in our sample, without certainty about the exact year of national implementation. Because of this limited and inconsistent documentation, screening uptake could not be incorporated into the models without compromising comparability.

### Role of the funding source

The funder had no role in the study design, data collection, data analysis, data interpretation, or writing of the report. The corresponding author had full access to all data and had final responsibility for the decision to submit for publication.

## Results

**Fig. 2:**
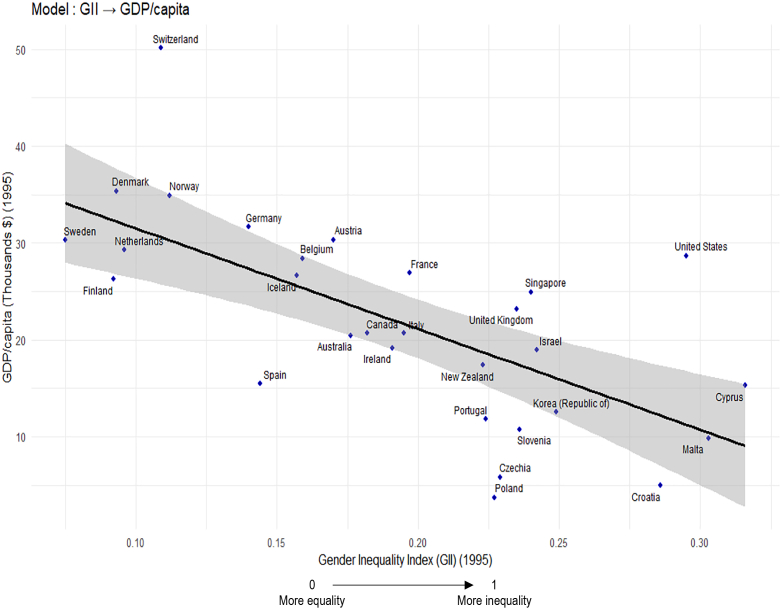
Association between Gender Inequality Index (1995) and GDP per Capita (1995). **Note:** Pearson correlation coefficient (r) between Gender Inequality Index (GII) and GDP per capita (expressed in thousands of US dollars): −0.670.

The final sample included 29 countries with high-quality cancer incidence and socioeconomic data (Fig. 1). Fig. 2 shows that Northern European countries had higher GDP and lower GII, while Eastern and Southern Europe had lower GDP and slightly higher GII, though all GII values were low (<0.35).

Table 1 shows that the lung cancer incidence sex ratio varied widely (median 1.90 [IQR 1.25–2.65]; range 0.94–4.14), while the colorectal cancer ratio was less variable (median 1.49 [IQR 1.35–1.75]; range 1.15–2.17). In 1995, median GDP/capita was 20.7 k$ (IQR 15.3–28.7 k$) and median GII was 0.20 (IQR 0.14–0.24).

**Table 1:** Sex ratio of incidence for lung and colorectal cancer, and distributions of GII and GDP per capita in the study population (N = 29).

Variable	Min	First quartile	Median	Mean	Third quartile	Max
Lung cancer sex ratio	0.935 (Iceland)	1.246	1.879	2.125	2.645	4.136 (Spain)
Colorectal cancer sex ratio	1.145 (Iceland)	1.354	1.490	1.533	1.754	2.172 (Croatia)
GDP per capita (1995, USD thousands)	3.687 (Poland)	15.261	20.680	21.870	28.691	50.114 (Switzerland)
GII (1995)	0.075 (Sweden)	0.144	0.195	0.193	0.236	0.316 (Cyprus)

### Lung incidence cancer sex ratio analysis

Regression results for lung cancer are shown in Table 2.

**Table 2:** Associations between gender inequality, economic context, and the sex ratio of lung cancer incidence: results from multiple regression models (GII, GDP per capita, and their interaction in Models 1–4) (N = 29).

Independent variable	Estimate (Beta)	p-value	Adjusted R^2^
M1_lung	0.492	0.004	0.235
GII			
M2_lung			0.354
GDP_category (Ind_High_GDP = reference)			
Ind_Medium_GDP	0.562	0.128	
Ind_Low_GDP	1.611	0.000	
M3_lung			0.332
GII	0.091	0.708	
GDP_category (Ind_High_GDP = reference)			
Ind_Medium_GDP	0.452	0.334	
Ind_Low_GDP	1.416	0.041	
M4_lung			0.439
GII	0.600	0.344	
GDP_category (Ind_High_GDP = reference)			
Ind_Medium_GDP	−0.134	0.869	
Ind_Low_GDP	−0.147	0.878	
GII∗GDP_category (Ind_High_GDP = reference)			
GII∗Ind_Medium_GDP	−0.893	0.199	
GII∗Ind_Low_GDP	0.482	0.549	

Note: These four sequential models follow standard analytical practice for assessing (1) the independent effect of each determinant, (2) their joint contributions, and (3) potential interactions. Their feasibility and robustness were verified through diagnostics and repeated estimations in sensitivity analyses.

Standardisation was applied to both variables (centring on the mean and dividing by the standard deviation).

Variance Inflation Factors–All multivariate models.

**Model 3**: GII = 2.61, GDP_Medium = 2.53, GDP_Low = 4.05.

**Model 4**: GII = 20.84, GDP_Medium = 8.98, GDP_Low = 10.09, GII × Medium = 6.41, GII × Low = 9.28.

**Model 3**: Moderate multicollinearity (VIF 2.5–5).

**Model 4**: Severe multicollinearity (VIF>5–10), rendering interaction estimates unreliable.

In Model 1 (GII), the association was positive and statistically significant (β = 0.492, p = 0.004), meaning that a one–SD increase in GII corresponded to a 0.492 SD increase in male over-incidence.

In Model 2 (GDP/capita), medium-GDP countries showed a non-significant increase in the sex ratio compared with high-GDP countries (β = 0.562, p = 0.128), whereas low-GDP countries had a significantly higher ratio (β = 1.611, p < 0.001).

In Model 3 (GII and GDP/capita), adding GDP/capita attenuated the associations. The effect of GII was no longer significant (β = 0.091, p = 0.708). Medium-GDP remained non-significant (β = 0.452, p = 0.334), and low-GDP was marginally significant (β = 1.416, p = 0.041).

In Model 4 (Model 3 + interaction), neither the main effects of GII (β = 0.599, p = 0.344) nor GDP/capita categories (medium: β = −0.134, p = 0.869; low: β = −0.147, p = 0.878), nor interactions (medium × GII: β = −0.893, p = 0.199; low × GII: β = 0.482, p = 0.549) were significant.

**Fig. 3:**
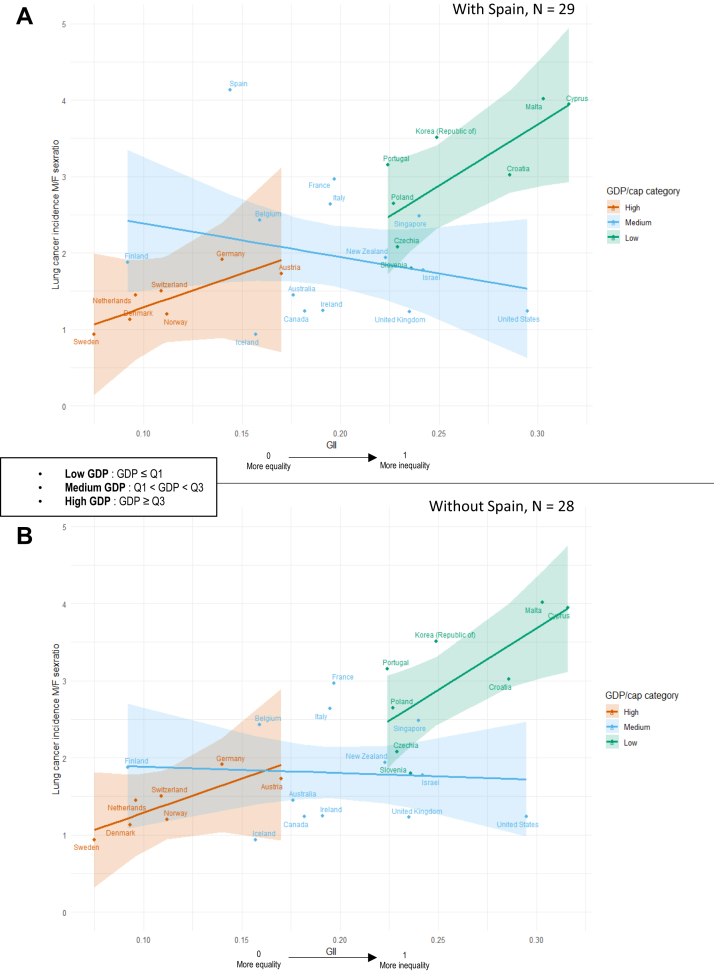
Association between Gender Inequality Index (1995) and lung cancer incidence sex ratio (M/F, <2022) in individuals over 70, stratified by GDP per capita categories (1995). Analyses with (A) and without (B) Spain. **Note:** These figures present the main analyses stratified by GDP per capita categories (1995). The tertiles used correspond to the empirical tertiles of GDP per capita (Q1 = 15,261; Q3 = 28,691). Sensitivity analyses using standardised continuous GDP were conducted; country classification remained stable and the results were similar.

Residual analysis (Appendix 1) identified Spain as the most influential country across in Models 2–4, while the United States was the most influential observation in Model 1. In the full sample (n = 29), Fig. 3A shows a positive association between GII and the lung cancer incidence sex ratio in low- and high-GDP countries. In contrast, a negative association was observed in medium-GDP countries (β = −0.893, p = 0.199; Table 2).

Sensitivity analyses excluding the most influential countries (Appendix 2) showed that overall patterns were stable, although effect sizes were attenuated and several associations lost statistical significance. After excluding Spain (n = 28; Fig. 3B), the negative slope in medium-GDP countries weakened (β = −0.656, p = 0.246; Appendix 2), indicating that this pattern was partly driven by Spain's leverage.

As complementary analyses, we examined the impact of the 2000 smoking-prevalence sex ratio on the relationship between GII, GDP, and lung cancer incidence sex ratio. Due to missing data, 25 countries were included. The sex ratio of tobacco consumption was strongly correlated with lung cancer incidence, particularly in wealthier and more gender-equal countries (Appendix 3).

In Model 1_bis (STable 2), the smoking-prevalence sex ratio was not significant (β = 0.306, p = 0.237), whereas GII remained significant (β = 0.521, p = 0.007), with a slightly stronger effect than in Model 1 (Table 2: β = 0.492, p = 0.004). When GDP was included, the smoking-prevalence sex ratio remained non-significant (β = 0.281, p = 0.097), while low-GDP countries continued to show significantly higher sex ratios (β = 1.343, p = 0.006).

The total effect of GII within each GDP category was calculated by combining main and interaction terms. In high-GDP countries, the effect was 0.599; in medium-GDP countries, the effect was negative (−0.294); and in low-GDP countries, it was stronger and positive (1.081), although both with high p-values indicating limited statistical robustness. After excluding Spain, the effect in low-GDP countries decreased substantially (0.482), while it remained unchanged in high-GDP countries (0.600) and became more negative in medium-GDP countries (−0.656), illustrating the strong leverage of Spain on these associations.

#### Colorectal incidence cancer sex ratio analysis

Regression analyses for colorectal cancer are presented in Table 3.

**Table 3:** Associations between gender inequality, economic context, and the sex ratio of colorectal cancer incidence: results from multiple regression models (GII, GDP per capita, and their interaction in Models 1–4) (N = 29).

Independent variable	Estimate (Beta)	p-value	Adjusted R^2^
M1_crc			0.020
GII	0.064	0.219	
M2_crc			0.363
GDP_category (Ind_High_GDP = reference)			
Ind_Medium_GDP	−0.030	0.770	
Ind_Low_GDP	0.360	0.003	
M3_crc			0.390
GII	−0.095	0.156	
GDP_category (Ind_High_GDP = reference)			
Ind_Medium_GDP	−0.084	0.507	
Ind_Low_GDP	0.563	0.004	
M4_crc			0.413
GII	0.187	0.307	
GDP_category (Ind_High_GDP = reference)			
Ind_Medium_GDP	−0.246	0.299	
Ind_Low_GDP	−0.319	0.256	
GII∗GDP_category (Ind_High_GDP = reference)			
GII∗Ind_Medium_GDP	−0.308	0.128	
GII∗ Ind_Low_GDP	−0.370	0.119	

Note: These four sequential models follow standard analytical practice for assessing (1) the independent effect of each determinant, (2) their joint contributions, and (3) potential interactions. Their feasibility and robustness were verified through diagnostics and repeated estimations in sensitivity analyses.

Standardisation was applied to both variables (centring on the mean and dividing by the standard deviation).

Variance Inflation Factors–All multivariate models.

**Model 3**: GII = 2.61, GDP_Medium = 2.53, GDP_Low = 4.05.

**Model 4**: GII = 20.84, GDP_Medium = 8.98, GDP_Low = 10.09, GII × Medium = 6.41, GII × Low = 9.28.

**Model 3**: Moderate multicollinearity (VIF 2.5–5).

**Model 4**: Severe multicollinearity (VIF>5–10), rendering interaction estimates unreliable.

In Model 1 (GII), the association was near zero and not significant (β = 0.064, p = 0.219).

In Model 2 (GDP/capita), compared to high-GDP countries, medium-GDP countries showed a flat and non-significant association (β = −0.030, p = 0.770), indicating little difference in the sex ratio of incidence. In contrast, low-GDP countries had a significantly higher sex ratio (β = 0.360, p = 0.003), reflecting substantially greater male over-incidence in these settings.

In Model 3 (GII and GDP/capita), the association between GII and sex ratio reversed direction, becoming negative but non-significant (β = −0.095, p = 0.156). The medium-GDP category also showed a negative, non-significant association (β = −0.084, p = 0.507), while the positive and significant association persisted in low-GDP countries (β = 0.563, p = 0.004).

In Model 4 (Model 3 + interaction), none of the main effect (GII: β = 0.187, p = 0.307; medium_GDP: β = −0.246, p = 0.299; low_GDP: β = −0.319, p = 0.256) nor the interaction terms (medium_GDP × GII: β = −0.308, p = 0.128; low_GDP × GII: β = −0.370, p = 0.119) were statistically significant.

Residual diagnostics identified four influential countries: Croatia, Malta, Spain, and Austria (Appendix 5). Robustness analyses excluding the most influential country in each model (Croatia for Model 1, Malta for Model 2, and Austria for Models 3 and 4) produced similar patterns, with only minor variations in effect sizes. Full coefficients, p-values, and adjusted R^2^ before and after exclusion are reported in Appendix 6.

**Fig. 4:**
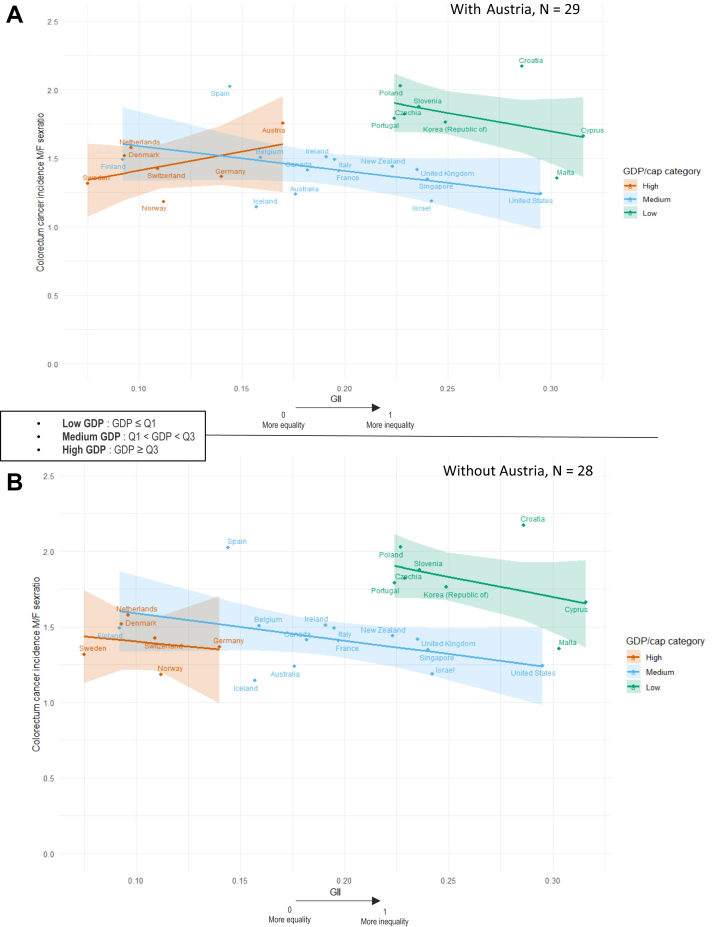
Association between Gender Inequality Index (1995) and colorectal cancer incidence sex ratio (M/F, <2022) in individuals over 70, stratified by GDP per capita categories (1995). Analyses with (A) and without (B) Austria. **Note:** These figures present the main analyses stratified by GDP per capita categories (1995). The tertiles used correspond to the empirical tertiles of GDP per capita (Q1 = 15,261; Q3 = 28,691). Sensitivity analyses using standardised continuous GDP were conducted; country classification remained stable and the results were similar.

Fig. 4A shows that, in the full sample (n = 29), the association between GII and the colorectal cancer incidence sex ratio varied by GDP/capita. In low-GDP countries, lower GII (greater gender equality) was associated with a smaller sex ratio, indicating a more balanced incidence between men and women. A similarly weak negative trend was observed in medium-GDP countries. In high-GDP countries, the association was positive, suggesting that higher GII may be linked to greater male over-incidence.

After excluding Austria (n = 28; Fig. 4B), the negative association in medium- and low-GDP countries persisted. In high-GDP countries, the association also became negative. These changes indicate that Austria exerted disproportionate influence on the observed associations, particularly within the high-GDP group. Removing Austria strengthened the negative association between gender equality and the sex ratio, suggesting that the initial pattern was partly driven by statistical leverage rather than a consistent trend across countries.

The total effect of GII in the full sample was 0.187 in high-GDP countries, −0.121 in medium-GDP countries, and −0.183 in low-GDP countries. After excluding Austria, the effect of GII became negative across all GDP categories (−0.088 in high-GDP, −0.012 in medium-GDP, and −0.183 in low-GDP countries), indicating that greater gender equality was consistently associated with a more balanced incidence between men and women across economic contexts (Appendix 6).

Similar analyses using continuous indicators for GII and GDP/capita produced nearly identical results for both lung and colorectal cancer (Supplementary Tables S1 and S2), further supporting the robustness of the findings.

## Discussion

This ecological study examined how structural gender inequality and macroeconomic context relate to sex differences in lung and colorectal cancer incidence across 29 high-income, gender-equal countries with high-quality cancer data. Our findings highlight distinct patterns for the two cancer sites, emphasising the need to integrate social and economic determinants into global cancer epidemiology.

For lung cancer, higher gender inequality was associated with greater male-to-female incidence ratios, particularly at the lowest and highest ends of the GDP spectrum. This suggests that economic context modulates the influence of gender inequality. Previous work^19^ has described a narrowing sex gap driven largely by gendered smoking patterns, yet our findings show that structural gender inequality remains an important correlate even after adjusting for historical tobacco use. This suggests that gender inequality shapes lung cancer disparities through mechanisms that extend beyond smoking, including occupational exposures, differential access to prevention, and structural constraints on health-seeking behaviours.

Occupational exposures account for an estimated 10–25% of lung cancer cases worldwide, with even higher attributable fractions in male-dominated sectors such as construction and mining.^20^ This offers one plausible pathway through which structural gender inequality influences lung cancer risk: men are disproportionately represented in jobs with high exposure to asbestos, silica, diesel exhaust and other carcinogens that substantially increase lung cancer incidence.^20^ These gendered differences in labour market participation are partly captured by the GII. In addition, gender inequality may shape access to health information, delays in diagnosis and the quality of care received, all of which can affect incidence patterns.

Economic context also modulated the relationship between GII and lung cancer. In low- and high-GDP settings within our sample, higher gender inequality was associated with greater male over-incidence, whereas in medium-GDP countries the association was negative but not robust. Sensitivity analyses showed that these variations were largely driven by influential observations, particularly Spain. Excluding this country attenuated effect sizes but did not change the overall direction. However, the strong correlation between gender inequality and economic development produced substantial multicollinearity in interaction models, limiting the precision of coefficient estimates and warranting cautious interpretation.

For colorectal cancer, GDP/capita emerged as the key determinant of the sex ratio of incidence. Higher-GDP countries exhibited narrower sex gaps, likely reflecting better access to screening, stronger healthcare systems, improved early detection, and healthier living conditions. This aligns with evidence showing that organised colorectal cancer screening is more widely implemented in high-income countries.^21^^,^^22^ However, variations in coverage, participation, and gender-specific barriers can still lead to unequal outcomes.^13^ For example, in France, participation is only 34.3% well below the 65% target set by health authorities, with slightly higher rates among women (35.7%) than men (33.5%).^23^ In the United Kingdom, participation is substantially higher, yet gender differences are larger, with 60.9% of women participating compared with 55.5% of men.^24^ Masculinity norms may negatively affect preventive healthcare behaviours, including participation in colorectal cancer screening.^25^ Conversely, gender equality has been associated with healthier behaviours among men,^26^ suggesting that environments with higher gender inequalities may reinforce disparities at multiple levels.^27^

Our analyses showed that the association between GII and the sex ratio of colorectal cancer incidence was weaker and more context-dependent, becoming borderline significant only when stratifying by economic level. This likely reflects the less gender-specific nature of colorectal cancer risk, which is increasingly influenced by metabolic and lifestyle-related factors —obesity, physical inactivity and diet—that have become more similar between men and women in high-income settings, contributing to a convergence in risk profiles.^28^ As a result, any structural influence of gender inequality is expected to be subtle. Nevertheless, stratified and sensitivity analyses revealed that its direction varied across GDP strata in the full sample but became consistently negative after removing Austria, an influential outlier. This suggests that greater gender equality may be associated with a more balanced incidence between men and women, although the effect sizes remained small.

To clarify why GII showed only limited associations for colorectal cancer, we developed a conceptual framework (Appendix 7) illustrating what the GII captures—and what it does not. Importantly, in this exploratory ecological analysis, behavioural factors such as alcohol consumption, diet, obesity, and screening uptake are considered mediators rather than confounders (Appendix 7). While we would have liked to include additional social and behavioural data to further unpack gender and economic inequalities, these variables do not bias the association between GII and cancer incidence in our models. The GII reflects only a subset of structural gender mechanisms and does not fully account for metabolic, behavioural, or healthcare-related pathways critical for colorectal cancer. This framework shows that the GII reflects only part of the structural and behavioural pathways relevant to colorectal cancer, which may explain the limited associations observed.

Beyond these statistical and contextual considerations, it is crucial to recognise that gender is not merely a demographic but a social construct shaping health trajectories across the life-course. From early childhood, gendered norms influence behaviours, opportunities and access to resources, including healthcare.^6^ By using early measures of gender inequality and economic development (1995), our analysis aimed to reflect this life-course perspective and capture the delayed structural effects on cancer incidence in older adults.

Even in high-income countries, where smoking and alcohol consumption have become more similar between men and women, structural gender inequalities may persist through differences in access to health information, screening and early detection. Such mechanisms are better captured by structural indices like the GII than by behavioural variables alone. As highlighted by Sen and Östlin (2008), gender norms and roles continue to influence health-seeking behaviours and the quality of care even in universal healthcare systems.^29^ These findings reinforce that gender inequality, when intersecting with other axes of marginalisation, can amplify disparities within well-resourced systems.

As an ecological exploratory study, residual confounding is expected, and the results cannot be interpreted causally. National-level indicators (GII and GDP per capita) reflect structural conditions and therefore do not capture individual-level mechanisms. In addition, gender inequality and economic development are strongly correlated at the country level. Although multicollinearity was moderate in models including only main effects, it became substantial in interaction models, limiting the ability to disentangle their independent contributions. The attenuation of GII coefficients after adjustment for GDP therefore likely reflects their shared structural variance rather than the absence of any relationship. The present models are thus better suited to identifying combined structural gradients than fully independent effects.

To ensure high data quality, we restricted the sample to countries with the most reliable cancer registry data, which resulted in a smaller but more robust analytical sample. Cancer incidence estimates depend on registry coverage and quality, which vary across countries, and differences in screening coverage may also affect detection and contribute to heterogeneity.

Sex-specific organised colorectal cancer screening uptake could not be included because comparable international data were available for only 14 of the 29 countries. Incorporating these data would have substantially reduced sample size and compromised comparability. Exploratory comparisons showed no clear difference in incidence sex ratios between countries with and without organised screening programmes, but heterogeneity in implementation timing and data quality limits interpretability (Supplementary Figure). Comparable international data on major colorectal cancer risk factors (e.g., alcohol consumption, dietary patterns) were also unavailable. Although international nutritional and behavioural databases exist, their incomplete coverage, lack of harmonised sex-disaggregated indicators and temporal misalignment precluded their inclusion in a unified multi-country ecological model.

The Gender Inequality Index (GII) facilitates international comparisons but does not capture all structural dimensions of gender that may influence health. Similarly, GDP per capita—used as a proxy for economic development—does not reflect actual resource allocation or equity in access to care. To account for economic context, GDP per capita was categorised into three groups (low, medium, high), which improves adjustment and comparability with the GII.^11^ However, discretizing GDP may lead to some loss of information, although this approach is widely used to reveal non-linear relationships and facilitate interpretation in international research.^10^

Despite these limitations, this study has several strengths. It is, to our knowledge, the first ecological analysis to explore how gender inequality and economic context interact to shape inequalities in incidence for the two deadliest non-sex-specific cancers. By combining high-quality cancer registry data with global indicators and adopting a life-course perspective using early exposure indicators, we propose a structural approach that remains rare in social cancer epidemiology. Stratified and sensitivity analyses—as well as consistent results obtained using continuous indicators for GII and GDP per capita—support the robustness of our findings. By identifying structural patterns in cancer incidence sex ratios across countries, this study provides a contextual framework for both population-based and clinical research, supporting future investigations that integrate biological, behavioural and social determinants as potential mechanisms through which gender and economic cancer inequalities are constructed.

Our findings underscore the need for global cancer monitoring to integrate not only behavioural and biological data but also social and economic structural indicators such as the GII and GDP per capita. Future research should include both sex assigned at birth and gender as distinct variables, and incorporate gender analyses to better understand and reduce cancer disparities.^30^

This exploratory study provides essential and still under-explored insights into how structural gender inequalities and economic context influence sex-specific cancer patterns, offering a framework to inform global cancer surveillance and guide more equitable prevention strategies in a socially diverse world.

## Contributors

AM, LB, PG, MKI, CD and SL conceived the study. AM conducted the statistical analyses. AM and LB verified the underlying data. AM draughted the manuscript. All authors contributed to the interpretation of the results and critically revised the manuscript for important intellectual content. All authors approved the final version of the manuscript.

## Data sharing statement

All data used in this study are publicly available. Cancer incidence data were obtained from the GLOBOCAN 2022 database. Gender inequality indicators were obtained from the United Nations Development Programme, and economic indicators from the World Bank. Additional behavioural data were obtained from the WHO Global Health Observatory. These datasets can be accessed directly from the respective institutional websites.

## Declaration of interests

The authors declare no competing interests.

## Appendix A Supplementary data

Supplementary Meterial
