# Supplementary material for: Sex ratio disparities in the two most common cancers worldwide: an exploratory analysis using GLOBOCAN 2022 data, gender inequalities, and economic indicators
Source: eClinicalMedicine. 2026 Apr 2;94:103855. doi: 10.1016/j.eclinm.2026.103855 (PMC13084316; doi:10.1016/j.eclinm.2026.103855)
Supplement: Supplementary Meterial [file mmc1.pdf]

Supplementary material

Supplementary material Table 1 : Associations between gender inequality, economic context, and the sex ratio of lung cancer incidence: results from multiple regression models (GII, GDP per capita in continuous, and their interaction in Models 1–3) (N=29).

Independent variable	Estimate (Beta)	P-value	Adjusted R2
M2_lung_:			0.280
GDP	-0.531	0.002	
M3_lung_:			0.292
GII	0.247	0.240	
GDP	-0.365	0.088	

Supplementary material Table 2 : Associations between gender inequality, economic context, and the sex ratio of colorectal cancer incidence: results from multiple regression models (GII, GDP per capita in continuous, and their interaction in Models 1-3) (N=29).

Independent variable	Estimate (Beta)	P-value	Adjusted R2
M2_crc			0.332
GDP	-0.162	0.001	
M3_crc			0.359
GII	-0.081	0.155	
GDP	-0.216	0.001	

Supplementary figure 1 : Relationship between sex-ratio of colorectal cancer and GDP interaction by Gender Inequality index (GII) (1995). Comparison between countries with and without

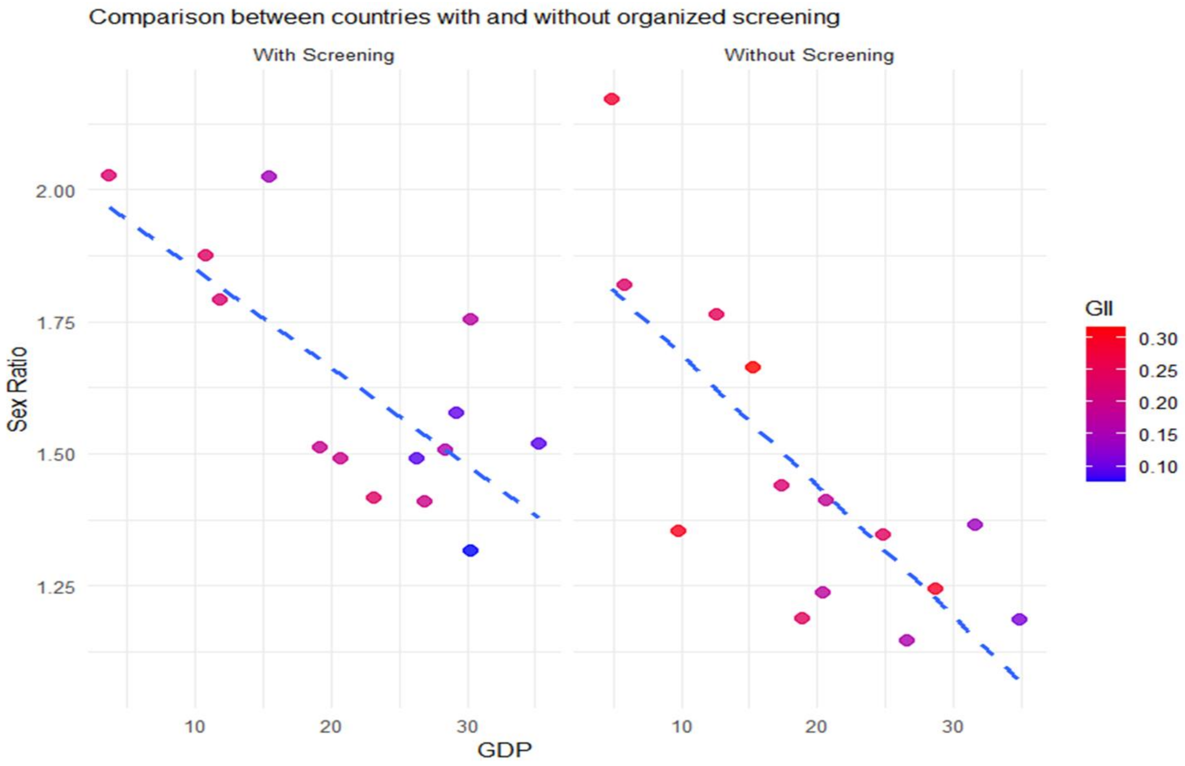

Note : This figure shows the relationship between GDP per capita (horizontal axis) and the sex ratio of colorectal cancer incidence (vertical axis), that is, the ratio of incidence rates in men to those in women.

Each point represents a European country, coloured according to its Gender Inequality Index (GII): the redder the colour, the greater the inequality.

The two graphs compare countries with organized screening programs (on the left, 14 countries : Austria, Belgium, Denmark, Finland, Ireland, Italy, Netherlands, Poland, Portugal, Slovenia, Spain, Sweden, United Kingdom, France) and those without organized screening (on the right, 15 countries).
