# Supplementary material for: Sex ratio disparities in the two most common cancers worldwide: an exploratory analysis using GLOBOCAN 2022 data, gender inequalities, and economic indicators
Source: eClinicalMedicine. 2026 Apr 2;94:103855. doi: 10.1016/j.eclinm.2026.103855 (PMC13084316; doi:10.1016/j.eclinm.2026.103855)
Supplement: Appendix [file mmc2.pdf]

Appendix 1 : Diagnostic plots for lung cancer models M1 to M4.

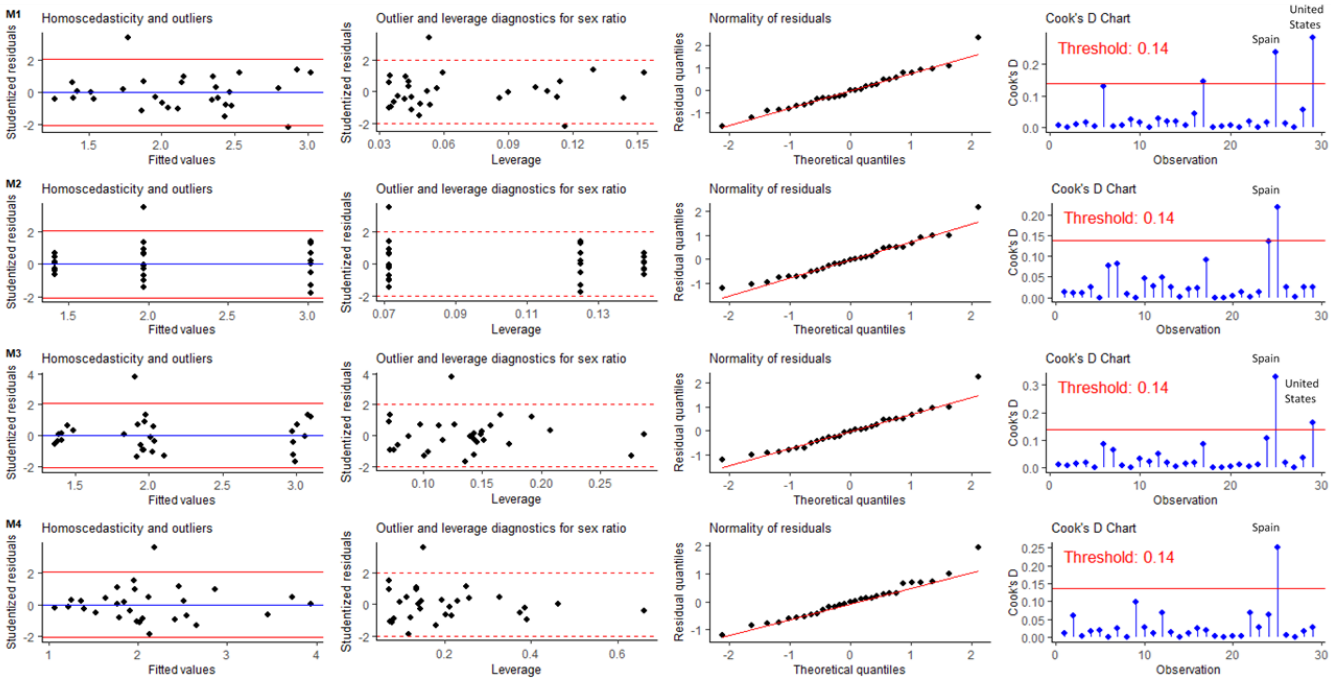

Note : For each model, from left to right: (1) studentized residuals versus fitted values (homoscedasticity and outliers), (2) leverage and outlier diagnostics, (3) Q-Q plot for normality of residuals, and (4) Cook's distance for influential observations. The red lines indicate standard thresholds.

- United States (Model 1): Low male-to-female lung cancer incidence ratio (1.24) combined with relatively high GII (0.30) made it the most influential observation (Cook's distance >0.14).

- Spain (Models 2–4): High male-to-female lung cancer incidence ratio (4.14), medium-GDP country (15,471 USD) with very low GII (0.14) generated a negative slope in the medium-GDP stratum, also exceeding the Cook's distance threshold (>0.14).

Appendix 2: STable 1 : Impact of gender inequality and economic context on the lung cancer sex ratio with Spain (N=29) and without Spain (N=28).

**Independent variable**	Main analyses			Sensitive analyses		
	**Estimate (Beta)**	**P-value**	**Adjusted R^2**	**Estimate (Beta)**	**P-value**	**Adjusted R^2**
M1_lung_:			0.235	Without United States (N=28)		
GII	0.492	0.004		0.592	0.001	0.335
M2_lung_:			0.354	Without Spain (N=28)		0.479
GDP_category (Ind_High_GDP = reference)						
Ind_Medium_GDP	0.562	0.128		0.395	0.204	
Ind_Low_GDP	1.611	0.000		1.611	0.000	
M3_lung_:			0.332	Without Spain (N=28)		
GII	0.091	0.708		0.269	0.190	0.497
GDP_category (Ind_High_GDP = reference)						
Ind_Medium_GDP	0.452	0.339		0.055	0.889	
Ind_Low_GDP	1.416	0.041		1.032	0.069	
M4_lung_:			0.439	Without Spain (N=28)		
GII	0.599	0.344		0.600	0.245	0.578
GDP_category (Ind_High_GDP = reference)						
Ind_Medium_GDP	-0.133	0.869		-0.304	0.645	
Ind_Low_GDP	-0.147	0.878		-0.147	0.850	
GII*GDP_category						
GII*Ind_Medium_GDP	-0.893	0.199		-0.656	0.246	
GII* Ind_Low_GDP	0.482	0.549		0.482	0.460	

Appendix 3 : STable 2 : Impact of sex ratio of consumption of tobacco on the association between gender inequality, economic context lung cancer sex ratio (N=25)

**Independent variable**	**Estimate (Beta)**	**P-value**	**Adjusted R^2**
M1_bis :			0.366
GII	0.521	0.007	
Sexratio of tobacco	0.306	0.237	
M2_bis :			0.362
GDP_category (Ind_High_GDP = reference)			
Ind_Medium_GDP	0.5471	0.175	
Ind_Low_GDP	1.343	0.006	
Sexratio of tobacco	0.281	0.097	
M3_bis :			0.351
GII	0.237	0.429	
GDP_category (Ind_High_GDP = reference)			
Ind_Medium_GDP	0.338	0.482	
Ind_Low_GDP	0.873	0.246	
Sexratio of tobacco	0.234	0.191	
M4_bis:			0.427
GII	0.643	0.329	
GDP_category (Ind_High_GDP = reference)			
Ind_Medium_GDP	-0.208	0.802	
Ind_Low_GDP	-0.339	0.730	
GII*GDP_category			
GII*Ind_Medium_GDP	0.261	0.130	
GII* Ind_Low_GDP	-0.908	0.235	
Sexratio of tobacco	0.330	0.692	

Appendix 4 : Relationship between the sex ratio (M/F) of tobacco consumption (2000) and the sex ratio (M/F) of lung cancer incidence (2022).

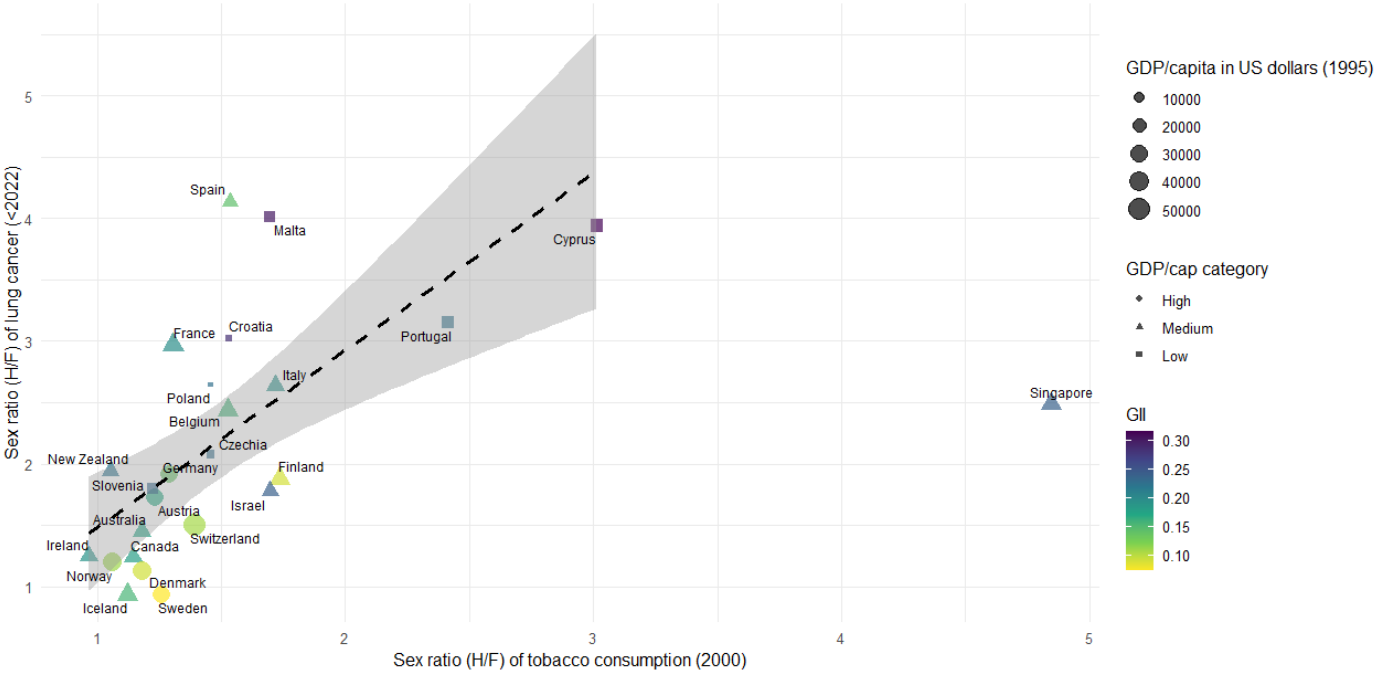

Note : Each point represents a country; point size is proportional to GDP per capita (1995), color indicates the Gender Inequality Index (GII), and shape denotes the GDP per capita category. The dashed line shows the linear regression (95% CI shaded); Singapore was excluded from the regression due to its outlier value.

Appendix 5 : Diagnostic plots for colorectal cancer models M1 to M4.

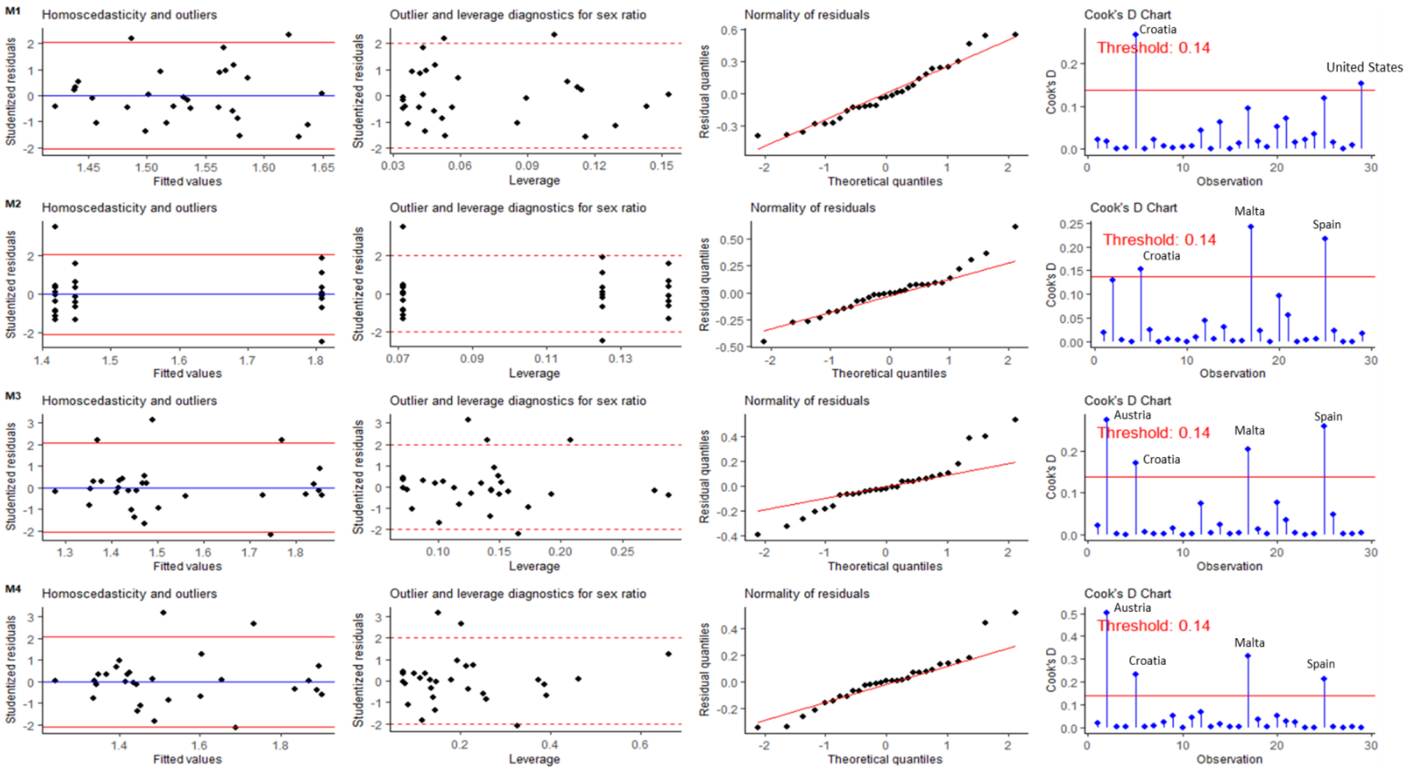

Note: For each model, from left to right: (1) studentized residuals versus fitted values (homoscedasticity and outliers), (2) leverage and outlier diagnostics, (3) Q-Q plot for normality of residuals, and (4) Cook's distance for influential observations. The red lines indicate standard thresholds.

- Austria (Models 3–4): Highest male-to-female colorectal cancer incidence ratio (1.75) within the high-GDP group (30,326 USD) and low GII (0.17), exceeding the Cook's distance threshold (>0.14).

Appendix 6 : STable 3 : Impact of gender inequality and economic context on the colorectal cancer sex ratio with all countries (N=29) and without influent countries (N=28).

**Independent variable**	Main analyses			Sensivity analyses		
	**Estimate (Beta)**	**P-value**	**Adjusted R2**	**Estimate (Beta)**	**P-value**	**Adjusted R2**
<u>M1_crc</u>				Without Croatia (N=28)		
GII	0.064	0.219	0.020	0.033	0.497	-0.020
<u>M2_crc</u>				Without Malta (N=28)		
GDP_category (Ind_High_GDP = reference)			0.363			0.477
Ind_Medium_GDP	-0.030	0.770		-0.029	0.749	
Ind_Low_GDP	0.360	0.003		0.425	0.000	
<u>M3_crc</u>			0.390	Without Austria (N=28)		
GII	-0.095	0.156		-0.132	0.045	0.477
GDP_category (Ind_High_GDP = reference)						
Ind_Medium_GDP	-0.084	0.507		0.199	0.132	
Ind_Low_GDP	0.563	0.004		0.714	0.000	
<u>M4_crc</u>			0.413	Without Austria (N=28)		
GII	0.187	0.307		-0.088	0.757	0.434
GDP_category (Ind_High_GDP = reference)						
Ind_Medium_GDP	-0.246	0.299		0.141	0.717	
Ind_Low_GDP	-0.319	0.256		0.706	0.099	
GII*GDP_category						
GII*Ind_Medium_GDP	-0.308	0.128		-0.032	0.913	
GII* Ind_Low_GDP	-0.370	0.119		-0.095	0.766	

<u>Note</u>: STable 3 reports the main and sensitivity analyses evaluating the associations between gender inequality (GII), economic context (GDP category), and the sex ratio of colorectal cancer incidence. Sensitivity analyses were conducted by removing countries identified as influential in the diagnostic assessments.

In Model M1 (excluding Croatia), the adjusted R^2 becomes negative (–0.020). This is statistically possible and simply indicates that the predictor (GII) does not improve model fit compared with the null model, reflecting the absence of association in this restricted sample.

Overall, the differences between adjusted R^2 values in the main and sensitivity analyses are modest and do not change the interpretation of the results. Conclusions remain consistent across the full sample and the reduced datasets.

Appendix 7 : Conceptual DAG illustrating the pathways potentially linking gender inequality, economic context and behavioural, social and health-related determinants to the colorectal cancer incidence sex ratio.

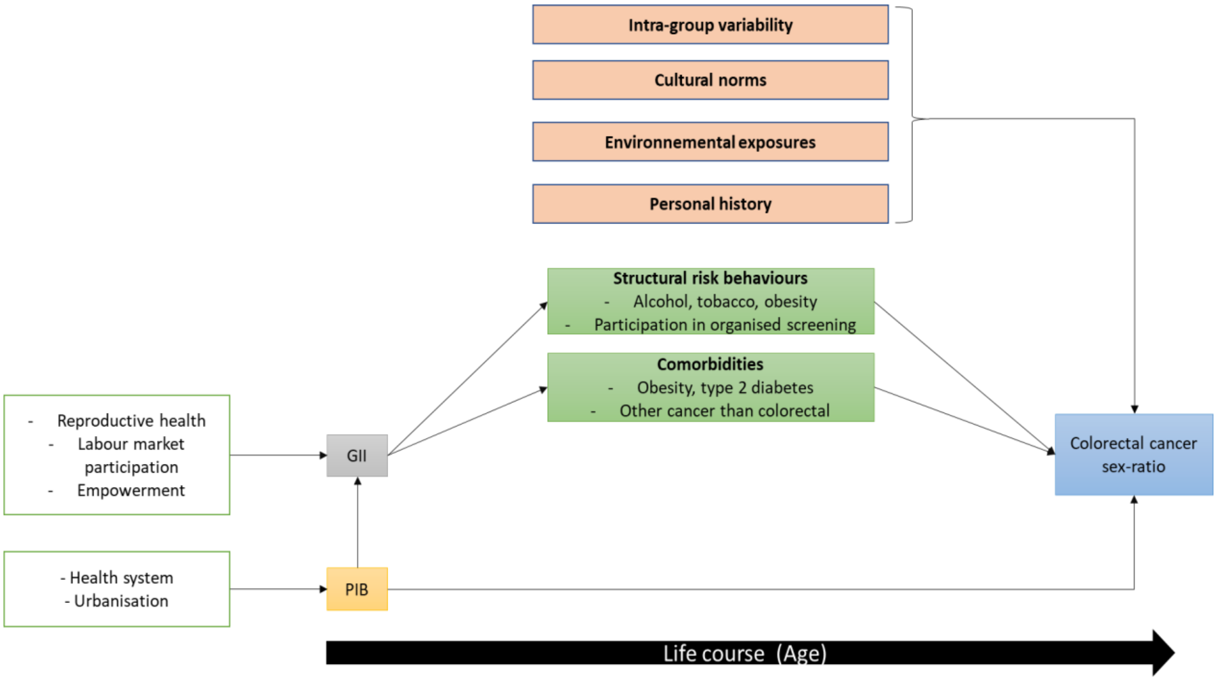

**Legend :**			
	Captured by the GII	Explanatory variable	Causal pathway
	Not captured by the GII	Confounding factor	
	Potentially captured	Outcome variable	
